# Risk assessment of an Aedes flavivirus and its effect on pathogenic flavivirus replication in mosquitoes

**DOI:** 10.1186/s13071-025-06711-4

**Published:** 2025-03-05

**Authors:** Yumei Fu, Wan Zhao, Shaohui Wu, Jinqian Li, Qing Liu, Feng Jiang, Hong Lu, Le Kang, Qianfeng Xia, Feng Cui

**Affiliations:** 1 State Key Laboratory of Integrated Management of Pest Insects and Rodents, Institute of Zoology, Chinese Academy of Sciences, Beijing, 100101 China; 2 NHC Key Laboratory of Tropical Disease Control, School of Tropical Medicine, Hainan Medical University, Haikou, 571199 Hainan China; 3 University of Chinese Academy of Sciences, Beijing, 100049 China

**Keywords:** Insect-specific flavivirus, Aedes flavivirus, *Aedes albopictus*, *Aedes aegypti*, *Culex quinquefasciatus*, Mammalian cells, Zika virus, Dengue virus

## Abstract

**Background:**

Mosquitoes are efficient vectors of medically significant flaviviruses and serve as hosts for insect-specific flaviviruses (ISFs). Aedes flavivirus (AEFV) is a classical ISF. Given the increasing discovery of ISFs, it is urgent to evaluate the potential risk of ISFs to human health as well as their impact on the transmission of pathogenic flaviviruses.

**Methods:**

We isolated a strain of AEFV from wild *Aedes albopictus* populations in Hainan Province, China, using iodixanol density-gradient ultracentrifugation. The infection of the AEFV Hainan strain in *Aedes*, *Culex*, and four mammalian cell lines was investigated using fluorescence in situ hybridization (FISH) assays, and relative and absolute quantitative polymerase chain reaction (qPCR). Whether AEFV alters the vector competence of *Ae. albopictus* for pathogenic arboviruses and the underlying immune mechanisms were explored.

**Results:**

The AEFV Hainan strain showed close genetic similarity to strains from Yunnan province of China, Thailand, and Peru. This strain was capable of infecting *Ae. albopictus* and *Ae. aegypti* but not *Culex quinquefasciatus*. Cell entry was the critical barrier for AEFV infection in *Cx. quinquefasciatus* cells. The infection risk of the AEFV Hainan strain in four mammalian cells (BHK-21, Vero, 293 T, and HeLa) was quite low due to the failure of cell entry or extremely limited replication. Prior infection of AEFV was detrimental to the replication of Zika virus and dengue virus serotype 2 in *Ae. albopictus* through activation of the Janus kinase/signal transducer and activator of transcription, Toll, or RNA interference pathway.

**Conclusions:**

Our work excludes the risk of the AEFV Hainan strain to human health and highlights its potential as an immune inducer to sabotage *Aedes* mosquito ability for viral transmission.

**Graphical Abstract:**

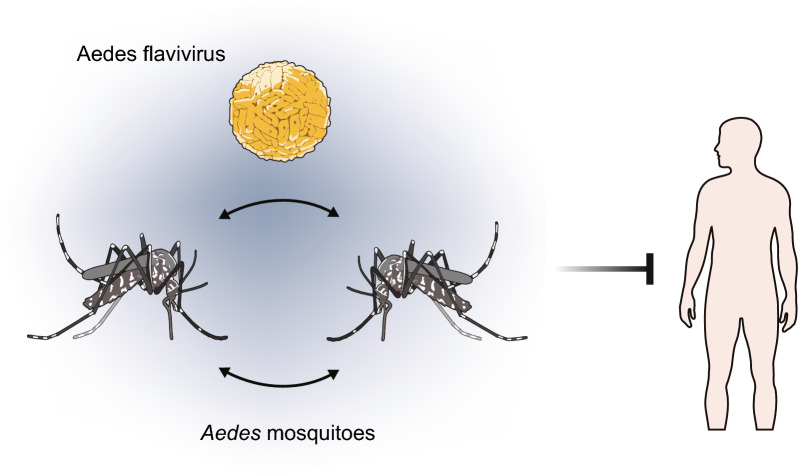

**Supplementary Information:**

The online version contains supplementary material available at 10.1186/s13071-025-06711-4.

## Background

Flaviviruses are positive-sense single-stranded RNA viruses predominantly transmitted by arthropods, including mosquitoes and ticks. *Aedes albopictus* and *Aedes aegypti* (Diptera: Culicidae) are typical mosquito vectors that efficiently transmit medically significant flaviviruses, such as dengue virus (DENV), West Nile virus (WNV), and Zika virus (ZIKV), causing a wide spectrum of clinical manifestations [1]. Besides these well-known pathogenic flaviviruses, mosquito-borne flaviviruses also include insect-specific flaviviruses (ISFs), which naturally replicate in mosquito cells but do not replicate in vertebrate cells [2]. ISFs are categorized into two distinct groups: classical insect-specific flaviviruses (cISFs), such as Aedes flavivirus (AEFV), and dual-host-affiliated insect-specific flaviviruses (dISFs), such as Donggang virus [3, 4]. Evolutionary studies have hypothesized that ISFs might be the ancestral forms from which the vertebrate-infecting flaviviruses evolved [5, 6], implying that ISFs may retain the potential to infect mammals. Over the past two decades, there has been a dramatic increase in reports of ISFs [3]. Timely detection, monitoring, and early warning of ISFs are effective strategies to prevent potential infectious diseases and protect human health.

As a typical member of cISFs, AEFV has been widely detected in wild female *Ae. albopictus* and *Aedes scapularis* across various regions, including the USA [7], Japan [3, 8], Peru [9], Thailand [10], and Italy [11]. AEFV exhibits high prevalence in *Aedes* and *Toxorhynchites* mosquito species, while *Anopheles* and *Culex* species are less permissive [10]. Additionally, AEFV is maintained in populations through vertical transmission [10]. However, the US, Japanese, and Peruvian strains of AEFV do not replicate in mammalian Vero or BHK-21 cells [7, 9, 12], and intracerebral inoculation of the US strain into newborn mice fails to produce observable illness [7]. Despite the progress made in understanding AEFV, the AEFV infection risk to mammals cannot be entirely ruled out, given the high frequency of viral mutations. Moreover, ISFs are capable of altering the mosquito’s susceptibility to pathogenic arboviruses [13]. The effects of AEFV on the replication and transmission of pathogenic arboviruses remain elusive.

In this study, we isolated a strain of AEFV from wild *Ae. albopictus* populations in Hainan Province, China. The host range of mosquito species and risk of infection in mammalian cells for this strain were evaluated. Additionally, the effects of AEFV on the replication of ZIKV and DENV2 (dengue virus serotype 2) in *Ae. albopictus* and the underlying immune mechanisms were explored.

## Methods

### Laboratory cultivation and wild collection of mosquitoes

Laboratory mosquito strains of *Ae. albopictus*, *Ae. aegypti*, and *Culex quinquefasciatus* were maintained in a climatic chamber under controlled conditions: 27 °C, 70% relative humidity, and a 16:8 h light/dark photoperiod. A 10% (w/v) sucrose solution was provided as a food source. Wild adults of *Ae. albopictus* were captured using mosquito traps in Wuzhishan city, Hainan Province, China.

### Mosquito and mammalian cell culture

C6/36, Aag2, and Cxq-1 were maintained at 28 °C in the following media: 1640 medium (Thermo Fisher, Waltham, MA, USA) for C6/36 cells, synthetic defined (SD) medium (Thermo Fisher) for Aag2 cells, and 199 medium (Sigma-Aldrich, St. Louis, MO, USA) for Cxq-1 cells. BHK-21, Vero, 293 T, and HeLa cells were maintained at 37 °C with 5% CO_2_ in Dulbecco’s modified Eagle medium (DMEM, Thermo Fisher). All media contained 10% fetal bovine serum (FBS), 100 U/ml of penicillin, and 100 μg/ml of streptomycin [14].

### Purification of AEFV Hainan strain and electron microscopy observation

Pools of five female *Ae. albopictus* from Wuzhishan city were homogenized in 350 μl of 1640 medium. The homogenates were clarified by centrifugation (13,000×*g* for 5 min at 4 °C) and filtered using a 0.22 μm filter. The filtered extracts were inoculated into a monolayer of C6/36 cells and incubated at 28 °C. At 5 days post-infection (dpi), cell supernatant was incubated with 10% (w/v) PEG 8000 overnight at 4 °C before centrifugation at 6500×*g* for 30 min. The pellet was solubilized with 1× phosphate-buffered saline (PBS) (pH 7.4) and applied in iodixanol density-gradient ultracentrifugation as described previously [15]. Each gradient was collected for AEFV *NS5* detection by real-time quantitative polymerase chain reactions (qPCR), and an AEFV positive gradient was applied in electron microscopy assays. The sample was loaded onto 200-mesh copper grids coated with a formvar/carbon film and glow-charged for 45 s. The grids were then stained with 1% uranyl acetate for 1 min and viewed under a transmission electron microscope (Tecnai Spirit) at 120 kV. This isolated virus was designated as the AEFV Hainan strain.

### Cell line infection with AEFV

Purified AEFV at a multiplicity of infection (MOI) of 6 × 10^6^ genome copies/μl was incubated with C6/36, Aag2, Cxq-1, BHK-21, Vero, 293 T, or HeLa monolayers in a 24-well plate, with each well containing 1 × 10^6^ cells. After incubation for 2 h, the viral suspension was removed. Cells were washed three times with 1× PBS buffer (pH 7.4) and then incubated with fresh 2% FBS basic medium at 28 °C. Culture supernatant and cell layers were collected after 1, 2, 3, 4, and 5 days and quantified by absolute and relative qPCR, respectively. A total of six biological replicates of supernatant and cell layers were prepared.

### Mosquito infection with AEFV

Three- to 5-day-old adult female mosquitoes were anesthetized on ice and intrathoracically injected with 150 nl of purified AEFV suspension at a MOI of 6 × 10^6^ genome copies/μl using a Nanoliter 2000 microinjector (World Precision Instruments, Sarasota, FL, USA). The whole bodies of mosquitoes were collected after 1, 3, and 5 days, and the viral loads were quantified by relative qPCR. Each mosquito served as one biological replicate. A total of 8 to 14 replicates of mosquitoes were prepared for quantification of viral loads in each group.

### Mosquitoes infected with AEFV and ZIKV or DENV2

Three- to 5-day-old adult female mosquitoes were intrathoracically injected with 150 nl of purified AEFV at a MOI of 6 × 10^6^ genome copies/μl. Control groups were injected with 2% 1640 medium. Mosquitoes were cultured for 5 days and starved for 12 h before being allowed to feed on a blood meal. The blood meal, consisting of a mixture of 50% fresh mouse blood and 50% viral suspension (ZIKV MOI 1 × 10^5^ focus-forming units [FFU]/ml or DENV2 MOI 1 × 10^8^ FFU/ml), was administered orally to the mosquitoes using a glass artificial feeding system with a Parafilm membrane. Mosquitoes were allowed to feed on the mixture for 2 h at 37 °C. Only fully engorged females were retained for further experiments. The whole bodies were collected at 3 and 7 dpi and salivary glands at 7 and 12 dpi. Each individual mosquito or two salivary glands represent one biological replicate. A total of 26 to 36 biological replicates for whole-body samples and 8 to 12 biological replicates for salivary gland samples were prepared for evaluating the relative RNA levels of *E* genes of ZIKV and DENV2. For quantifying the relative expression levels of protein inhibitor of activated signal transducer and activator of transcription (*PIAS*), *Cactus*, and *Dicer2*, 16 to 24 biological replicates were prepared.

### RNA extraction and cDNA synthesis

Total RNA from cell culture supernatant was extracted using the TIANamp Virus RNA Kit (Tiangen, Beijing, China). Total RNA from cell layers and individual mosquitoes was extracted using TRIzol reagent (Invitrogen, Carlsbad, CA, USA) according to the manufacturer’s instructions. A total of 2 μg or 4 μl of RNA was reverse-transcribed to complementary DNA (cDNA) using M-MLV reverse transcriptase (RT) and random primers (Promega, Madison, WI, USA) for further relative or absolute qPCR assays, respectively.

### Relative qPCR

Relative qPCR was performed to quantify the RNA levels of the AEFV *NS5* [10] and envelope glycoprotein (*E*) gene of ZIKV and DENV2 [16], and the transcript levels of *PIAS* (AALFPA_068822 in VectorBase), *Cactus* (AALF016282 in VectorBase), and *Dicer2* (AALF027719 in VectorBase) of *Ae. albopictus* on a LightCycler 480 Instrument II (Roche, Basel, Switzerland) using a LightCycler 480 SYBR Green I Master Mix (Roche). The mosquito *actin* gene of *Ae. albopictus* (GenBank accession no. DQ657949.1), *Ae. aegypti* (KY000701), and *Cx. quinquefasciatus* (XM_001847218.2), and the mammalian *actin* gene of hamster (NM_031144), African green monkey (XM_012087502.1), and human (NM_001101.5) were quantified as endogenous controls. RNA levels of viral genes or relative expression levels of mosquito genes were calculated based on the 2^−∆∆Ct^ method [17] and are reported as the mean ±  standard error (SE). At least six replicates were prepared. Primers used in this experiment are listed in Additional file 1: Table S1. All PCR products were sequenced for validation.

### Absolute qPCR

Viral titers in cell culture supernatant were evaluated by quantifying the copy numbers of the AEFV *NS5* gene, with primers listed in Additional file 1: Table S1. The amplified products were cloned into pLB vector (Tiangen) and transformed into competent *Escherichia coli* strain Trans T1 cells (TransGen, Beijing, China). Eight serial 10-fold dilutions of the recombinant plasmid were prepared, ranging from 1 ng/μl to 10^–7^ ng/μl. The standard curve was generated as described previously [15].

### Rapid amplification of cDNA ends (RACE)

The 5′ and 3′ ends of the AEFV genome were acquired using the SMARTer RACE 5′/3′ Kit (Takara, Mountain View, CA, USA) according to the manufacturer’s protocol. The nested PCR primers are listed in Additional file 1: Table S1. The amplification program was as follows: (1) five cycles of 94 °C for 30 s, 72 °C for 3 min, (2) five cycles of 94 °C for 30 s, 70 °C for 30 s, 72 °C for 3 min, and (3) 20 cycles of 94 °C for 30 s, 68 °C for 3 min. The amplified products were cloned into pLB vector and subjected to Sanger sequencing.

### Fluorescence in situ hybridization (FISH) assay

A 349-base-pair (bp) fragment from 502 nucleotides (nt) to 850 nt of the AEFV coat protein (*CP*) gene was cloned (primers in Additional file 1: Table S1) and served as a DNA template for synthesizing probes using the T7/SP6 RNA Transcription Kit (Roche) and Biotin RNA Labeling Mix (Roche) according to the manufacturer’s protocol. AEFV suspension at a MOI of 6 × 10^6^ genome copies/μl was incubated with cell monolayers for 2 h. After they were washed with 1× PBS buffer (pH 7.2) containing 0.025% trypsin, the infected cells were fixed with 4% (w/v) paraformaldehyde and digested by 20 ng/μl proteinase K to digestion at room temperature for 10 min. Hybridization with the probe (15–30 ng/μl) was performed at 37 °C overnight, followed by sequential washes in 2×, 1×, and 0.2× saline-sodium citrate (SSC). Biotin-Alexa 549 antibody was incubated for 2 h. The cytomembrane was stained with DiD perchlorate and the nucleus was stained with DAPI. Images were viewed under a confocal microscope (Leica).

### Plaque assay on ZIKV and DENV2

The ZIKV MR766 (GenBank accession no. HQ234498) and DENV2 NGC (GenBank accession no. AF038403.1) strains were passaged in C6/36 cells to generate high-titer stocks. The viral stock was then serially diluted in 10-fold increments with DMEM to create six concentrations. Each viral dilution was incubated with Vero cells for 2 h. The medium was then replaced with DMEM containing 1.2% methylcellulose plus 5% FBS. After incubation at 37 °C with 5% CO_2_ for 5 days, cells were fixed with 4% paraformaldehyde and stained with 1% crystal violet solution.

### Sequence alignment and phylogenetic analysis

The whole-genome sequences of 11 AEFV strains from Switzerland, Italy, the USA, Japan, Turkey, Thailand, Peru, Indonesia, and China’s Yunnan province were obtained from the National Center for Biotechnology Information (NCBI) and the Bacterial and Viral Bioinformatics Resource Center (https://www.bv-brc.org/). These AEFV genome sequences and the Hainan strain were aligned to construct a neighbor-joining phylogenetic tree using MEGA7 software with the pairwise deletion and p-distance model and bootstrap of 1000 replicates. The Culex flavivirus genome sequence served as an outgroup.

### Statistical analysis

All bar charts were generated using GraphPad Prism 10 software (GraphPad Software, San Diego, CA, USA) based on the original experimental data. Statistical differences were evaluated using Student’s *t*-test for two-group comparisons and one-way analysis of variance (ANOVA) followed by Tukey’s and Tamhane’s tests for multiple-group comparisons in SPSS 19.0.

## Results

### Distribution of Aedes flavivirus in mosquito species in Hainan Island

**Fig. 1:**
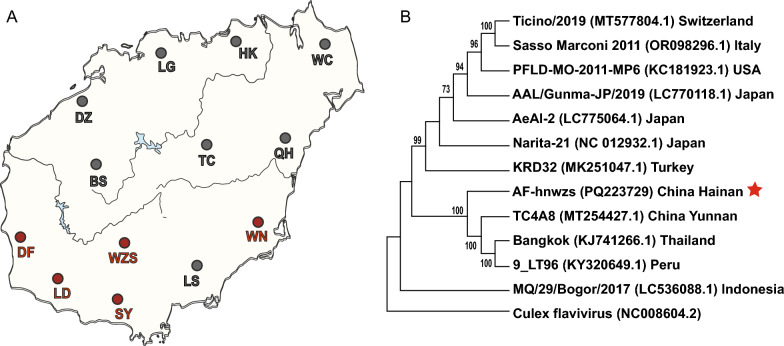
Distribution of AEFV in populations of *Ae. albopictus* in Hainan Island. **A** Geographical distribution of AEFV detected in *Ae. albopictus*. Red dots indicate areas where AEFV was detected, whereas gray dots represent areas where AEFV was not detected. The map is based on a standard geographical template obtained from the National Administration of Surveying, Mapping and Geoinformation with approval number GS (2019)3333. HK, Haikou; DF, Dongfang; LD, Ledong; WZS, Wuzhishan; SY, Sanya; WN, Wanning; LS, Lingshui; QH, Qionghai; TC, Tunchang; BS, Baisha; DZ, Danzhou; LG, Lingao; WC, Wenchang. **B** Neighbor-joining phylogenetic tree of AEFV identified in various regions based on viral genome sequences. The sequence of Culex flavivirus is used as the outgroup. Bootstrap values higher than 70% are shown at the nodes

AEFV sequences were identified based on the sequencing data from wild mosquitoes collected from 13 counties across Hainan Island between 2018 and 2020 [18]. Among the 15 mosquito species sampled, including nine *Culex*, four *Aedes*, one *Anopheles*, and one *Armigeres*, the AEFV fragments were detected exclusively in *Ae. albopictus* from five regions in the southern part of Hainan Island: Wuzhishan, Dongfang, Ledong, Sanya, and Wanning (Fig. 1A). Wuzhishan was the most prevalent area for AEFV. The full length of the AEFV genome was obtained using RT-PCR (reverse transcription-polymerase chain reaction) and RACE following Sanger sequencing. The 11,061-nt genome consists of a 10,026-nt open reading frame, a 96-nt 5′ untranslated region (UTR), and a 939-nt 3′ UTR (GenBank number no. PQ223729). Based on viral genomic sequence, neighbor-joining phylogenetic analysis revealed that this AEFV Hainan strain (named AF-hnwzs) shares close genetic similarity with strains from Yunnan province of China, Thailand, and Peru, while it is more divergent from another cluster that includes AEFV strains from Switzerland, Italy, the USA, Japan, and Turkey (Fig. 1B). Both clusters appear to have originated from an Indonesian AEFV strain (Fig. 1B).

### Mosquito host range of the AEFV Hainan strain

**Fig. 2:**
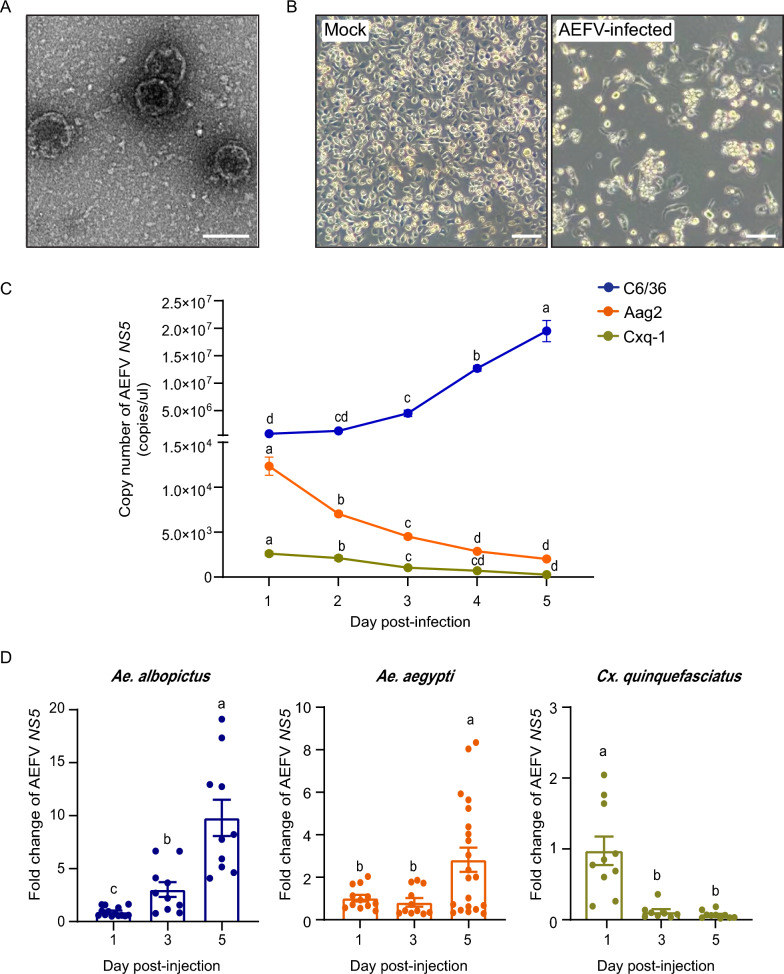
Mosquito host range of the AEFV Hainan strain. **A** Transmission electron micrograph of purified AEFV Hainan strain. Scale bar represents 50 nm. **B** The CPE of C6/36 cells infected with the AEFV Hainan strain at 4 dpi. Scale bars represent 50 μm. **C** Copy number of AEFV *NS5* in the supernatant of three mosquito cell lines infected with purified virions at 6 × 10^6^ copies/μl quantified by absolute qPCR. **D** Fold change of AEFV *NS5* in the whole bodies of three mosquito species injected with purified virions. The RNA levels of *NS5* relative to those of *actin* at 1 day post-injection are set as 1. Each dot represents one individual mosquito. For (**C**) and (**D**), the values are presented as mean ± SE, and different letters indicate statistically significant differences

The AEFV Hainan strain was isolated from wild females of *Ae. albopictus* using iodixanol density-gradient ultracentrifugation and cultured in the *Ae. albopictus* C6/36 cell line. Under electron microscopy, the virions appeared as round shapes with diameters of approximately 40–50 nm (Fig. 2A), consistent with previous reports [2, 7, 9, 11]. A clear cytopathic effect (CPE) was observed in C6/36 cells at 4 dpi, characterized by cell swelling, syncytia formation, vacuolation, and monolayer destruction (Fig. 2B). Purified virions were used to infect cells from other mosquito species, including *Ae. aegypti* Aag2 and *Cx. quinquefasciatus* Cxq-1 [19]. Viral titers in cell culture supernatant were quantified by measuring the copy number of the viral *NS5* gene using absolute qPCR. Unlike the significant increase in AEFV titers observed in the supernatant of C6/36 cells after 3 dpi, no increase was observed in the supernatant of Aag2 and Cxq-1 cells within 5 dpi (Fig. 2C). When purified virions were injected in female adult mosquitoes, viral loads, measured as the relative RNA level of *NS5*, increased over time in both *Ae. albopictus* and *Ae. aegypti* but not in *Cx. quinquefasciatus* within 5 days (Fig. 2D). Although the results were inconsistent for *Ae. aegypti* at the cellular and individual levels, we determined that *Ae. albopictus* and *Ae. aegypti* are natural hosts for the AEFV Hainan strain, while *Cx. quinquefasciatus* is not.

### Infection barriers of the AEFV Hainan strain in different mosquito species

**Fig. 3:**
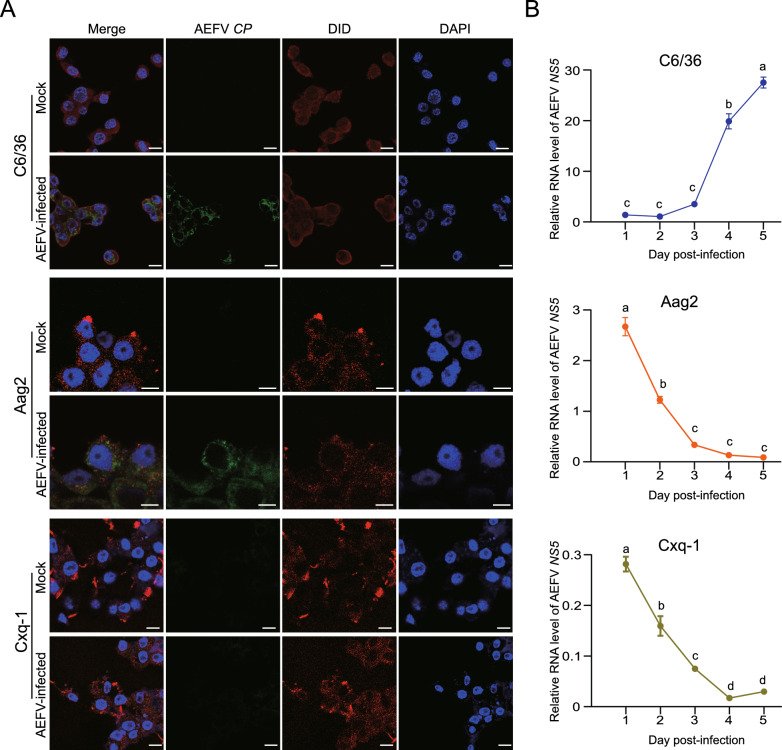
Infection barriers of the AEFV Hainan strain in different mosquito species. **A** FISH assays on three mosquito cell lines incubated with purified AEFV virions for 2 h using the biotin-labeled AEFV *CP* probe. Samples that were not infected with AEFV serve as the negative control (mock). DiD and DAPI were used to stain the cytomembrane and nucleus, respectively. Scale bars represent 20 μm. **B** The RNA levels of AEFV *NS5* relative to those of *actin* in three mosquito cells infected with purified virions. The values are presented as mean ± SE, and different letters indicate statistically significant differences

To clarify the barriers preventing AEFV infection in *Ae. aegypti* and *Cx. quinquefasciatus* cells, we first investigated the cell entry of AEFV within 2 h using biotin-labeled probes targeting the AEFV *CP* gene in FISH assays. As a positive control, AEFV was internalized into C6/36 cells with a high infectious rate of 91% (152 positive out of 167 cells) (Fig. 3A). Fluorescence signals of AEFV were also observed inside Aag2 cells, but the infectious rate was only 25.3% (21 positive out of 83 cells) (Fig. 3A). No fluorescence signals were detected in Cxq-1 cells (none positive out of 112 cells) (Fig. 3A). Viral replication was then evaluated by quantifying viral amounts in the cell layers using relative qPCR within 5 days after incubation with AEFV. The amount of AEFV gradually increased over time in C6/36 cells while sharply decreasing over time in Aag2 and Cxq-1 cells (Fig. 3B). These results showed that the cell entry efficiency and replication capacity of the AEFV Hainan strain are quite limited in *Ae. aegypti* cells, and the cell entry is the critical barrier for AEFV infection in *Cx. quinquefasciatus* cells.

### Risk assessment of the AEFV Hainan strain in different mammalian cells

**Fig. 4:**
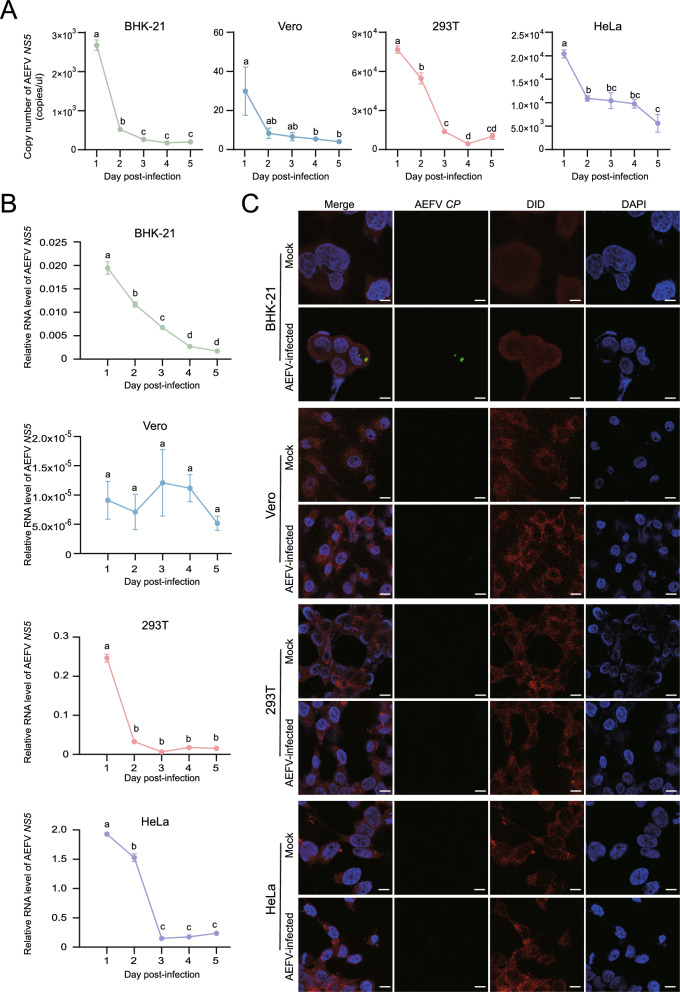
Risk assessment of the AEFV Hainan strain in different mammalian cells. **A** Copy number of AEFV *NS5* in the supernatant of four mammalian cell lines infected with purified virions at 6 × 10^6^ copies/μl quantified by absolute qPCR. **B** The RNA levels of AEFV *NS5* relative to those of *actin* in four mammalian cells infected with purified virions at 6 × 10^6^ copies/μl. For (**A**) and (**B**), the values are presented as mean ± SE, and different letters indicate statistically significant differences. **C** FISH assays on four mammalian cell lines incubated with purified AEFV virions at 6 × 10^6^ copies/μl for 2 h using the biotin-labeled AEFV *CP* probe. Samples that were not infected with AEFV serve as the negative control (mock). DiD and DAPI were used to stain the cytomembrane and nucleus, respectively. Scale bars represent 20 μm

To evaluate the infection risk of the AEFV Hainan strain in mammalian cells, we incubated AEFV suspension into hamster kidney (BHK-21), African green monkey kidney (Vero), human kidney epithelial (293 T), and human cervical carcinoma (HeLa) cells. Absolute qPCR results showed that the viral titers in the supernatant of these four mammalian cell lines decreased significantly over time within 5 days (Fig. 4A). Additionally, AEFV amounts within the cell layers of BHK-21, 293 T, and HeLa decreased or remained unchanged in Vero cells over the same period (Fig. 4B), indicating that AEFV did not replicate in any of the four mammalian cell lines. To investigate viral cell entry, FISH assays were conducted in the four cell lines after 2 h incubation with AEFV using biotin-labeled probes targeting the AEFV *CP* gene. Fluorescence signals were observed inside BHK-21 cells with a low infectious rate of 4.2% (4 positive out of 95 cells), but no signals were detected in Vero (0 positive out of 114 cells), 293 T (0 positive out of 125 cells), or HeLa (0 positive out of 117 cells) cells (Fig. 4C). These results demonstrated that the infection risk of the AEFV Hainan strain in these four mammalian cell lines is very low due to the failure of cell entry or extremely limited replication.

### AEFV inhibits the replication of pathogenic arboviruses in *Ae. albopictus*

**Fig. 5:**
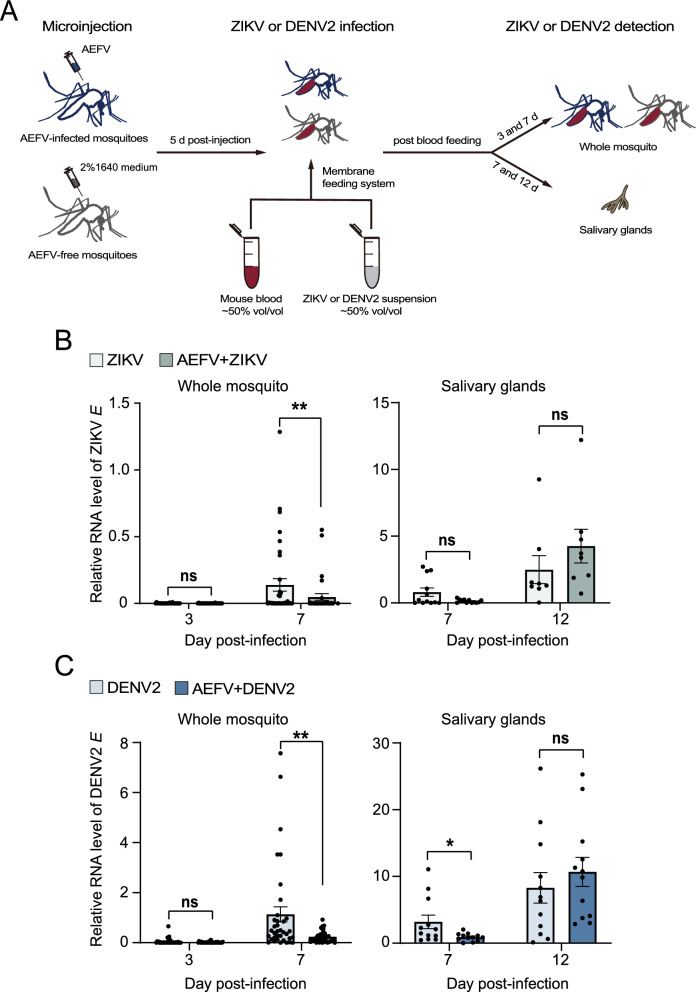
AEFV inhibits the replication of pathogenic arboviruses in *Ae. albopictus.* **A** Schematic of the experimental design. **B** The RNA levels of the ZIKV *E* gene relative to those of *actin* in the whole bodies and salivary glands of *Ae. albopictus* infected with ZIKV alone or ZIKV plus AEFV. **C** The RNA levels of the DENV2 *E* gene relative to those of *actin* in whole bodies and salivary glands of *Ae. albopictus* infected with DENV2 alone or DENV2 plus AEFV. For (**B**) and (**C**), each dot represents one individual mosquito or two salivary glands in one replicate. The values are presented as mean ± SE. ns, no statistically significant difference. *P < 0.05. **P < 0.01

*Aedes albopictus* is a major vector of pathogenic arboviruses such as ZIKV and DENV2. To determine whether AEFV alters the vector competence of *Ae. albopictus* for these two arboviruses, we injected female *Ae. albopictus* adults with the AEFV Hainan strain. Five days later, the mosquitoes were fed on blood containing ZIKV or DENV2 using a membrane blood-feeding system. The viral loads were quantified in the whole bodies at 3 and 7 dpi and in salivary glands at 7 and 12 dpi (Fig. 5A). Relative qPCR results showed that the titer of ZIKV, measured by the RNA level of the viral *E* gene, decreased by threefold in the whole bodies of mosquitoes at 7 dpi when pre-infected with AEFV (Fig. 5B). However, the ZIKV loads in salivary glands remained unchanged between the AEFV-free and AEFV-infected groups (Fig. 5B). Similar phenomena were observed for DENV2 infection. The viral loads of DENV2, measured by the RNA level of the viral *E* gene, dramatically decreased by fivefold at 7 dpi in the whole bodies of mosquitoes carrying AEFV compared to those without AEFV (Fig. 5C). Although a significant reduction of DENV2 in the salivary glands was observed at 7 dpi in the AEFV-infected group, there was no difference between the two groups at 12 dpi (Fig. 5C). These results demonstrated that the presence of AEFV is detrimental to the replication of ZIKV and DENV2 in *Ae. albopictus*, but has a limited impact on the transmission of these two arboviruses.

### AEFV activates antiviral responses in *Ae. albopictus*

**Fig. 6:**
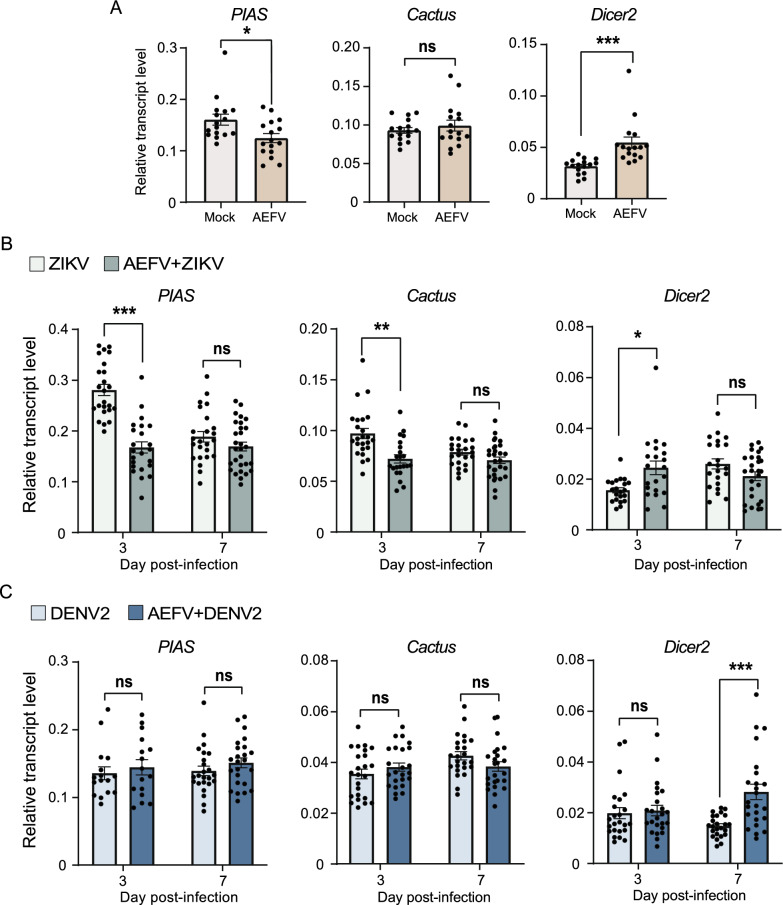
AEFV activates antiviral responses in *Ae. albopictus*. **A** Transcript levels of three immune genes relative to those of *actin* in *Ae. albopictus* injected with AEFV for 5 days. The mock group was injected with 2% 1640 medium. **B** Transcript levels of three immune genes relative to those of *actin* in *Ae. albopictus* infected with ZIKV alone or ZIKV plus AEFV. **C** Transcript levels of three immune genes relative to those of *actin* in *Ae. albopictus* infected with DENV2 alone or DENV2 plus AEFV. The experimental procedure is the same as shown in Fig. 5A. Each dot represents one individual mosquito. The values are presented as mean ± SE. ns, no statistically significant difference. *P < 0.05. **P < 0.01. ***P < 0.001

The Janus kinase/signal transducer and activator of transcription (JAK-STAT), Toll, and RNA interference (RNAi) pathways play essential antiviral roles in *Aedes* mosquitoes in defense against ZIKV and DENV infections [20]. To understand the immune mechanisms responsible for the negative impact of AEFV on the replication of ZIKV and DENV2 in *Ae. albopictus*, we measured the response to AEFV infection for *PIAS*, *Cactus*, and *Dicer2* in the JAK-STAT, Toll, and RNAi pathways, respectively. *PIAS* and *Cactus* function as negative regulators of their respective pathways. When the mosquitoes were inoculated with AEFV alone for 5 days, both the JAK-STAT and RNAi pathways were activated, as evidenced by the downregulated expression of *PIAS* and the upregulation of *Dicer2* (Fig. 6A). The Toll pathway did not seem to be influenced, as *Cactus* expression remained unchanged after AEFV infection (Fig. 6A). When the mosquitoes were pre-infected with AEFV for 5 days and then subsequently inoculated with ZIKV, the expression levels of *PIAS* and *Cactus* were downregulated and *Dicer2* was upregulated at 3 dpi compared to mosquitoes infected with ZIKV alone, though not at 7 dpi (Fig. 6B). These dates indicated that the JAK-STAT, Toll, and RNAi pathways were more active with the composite infection of AEFV and ZIKV than with ZIKV alone, leading to the suppression of ZIKV replication in the presence of AEFV. For the combined infection of AEFV and DENV2, only *Dicer2* was upregulated at 7 dpi compared to the infection of DENV2 alone (Fig. 6C). Therefore, the activation of the RNAi pathway likely accounts for the inhibition of DENV2 replication in mosquitoes pre-infected with AEFV.

## Discussion

Mosquitoes are one of the most important vectors for flavivirus transmission. Monitoring the content and types of flaviviruses in mosquitoes helps us to better prepare and warn against mosquito-borne diseases. In this study, we isolated an ISF, AEFV, from wild *Ae. albopictus* populations in Hainan Island. The AEFV Hainan strain replicated in *Ae. albopictus* and *Ae. aegypti* but not in *Cx. quinquefasciatus*. Although the infection risk of the AEFV Hainan strain in mammalian cells was quite low due to the bottleneck of cell entry and replication, it has the potential to interfere with the vector competence of *Ae. albopictus* for ZIKV and DENV through activation of antiviral response to inhibit the replication of the two pathogenic arboviruses in mosquitoes. These results suggest that AEFV could be used as an immune inducer to suppress pathogenic flavivirus loads in *Aedes* mosquitoes during viral disease outbreaks.

The AEFV Hainan strain has a similar host range to other known strains. Our finding shows that the AEFV Hainan strain can infect *Ae. albopictus* and *Ae. aegypti* but not *Cx. quinquefasciatus*, which aligns with previous records indicating that AEFV is prevalent in *Aedes* species while being incompatible with *Culex* species [10]. However, our study observed different outcomes between cell lines and live *Ae. aegypti*: while AEFV did not replicate in Aag2 cells within 5 days, it successfully infected adult *Ae. aegypti*, with viral loads increasing over time. This discrepancy can be attributed to the fact that AEFV may be restricted to specific tissues for replication. In addition, the AEFV Hainan strain does not infect mammalian cells such as BHK-21, Vero, 293 T, or HeLa. Similar evaluations have been described for other AEFV strains, such as the Japanese Narita-21 strain [12], US SPFLD-MO-2011-MP6 strain [7], and Peruvian 49_LT96 strain [9]. These findings demonstrate that the infection risk of these four AEFV strains to humans or mammals remains quite low, despite accumulating a few mutations, with nucleotide sequence identities ranging from 90 to 98% and amino acid sequence identities exceeding 97%. Additionally, different culturing temperatures may have influenced the host selection of the AEFV strains. Insect cells were cultured at 28 °C, while mammalian cells were cultured at 37 °C. This temperature difference could pose a huge gap for ISFs to cross the species barrier. For example, Rabensburg virus was incapable of infecting Vero and E6 cells at 37 °C, but it exhibited efficient infection and replication in these cells when cultured at 28 °C [21].

ISFs exhibit restricted infection in mammals at multiple stages of the infection cycle. For example, Aripo virus is endocytosed into Vero cells but fails to replicate [22]. Binjari virus (BinJV) does not replicate in A549 cells because its genome contains a higher frequency of CpG dinucleotides than vertebrate-infecting flaviviruses, which interact with the zinc-finger antiviral protein [23]. BinJV replication in BSR cells is also temperature-sensitive and restricted by inefficient cell entry and susceptibility to antiviral responses [24]. Niénokoué virus faces infectious restrictions at the viral assembly and release stages in BHK cells [25]. Similarly, AEFV infection in mammalian cells is restricted at both cell entry and replication stages. Host receptors play key roles in the cell entry of flaviviruses. Various molecules, such as heparin sulfate [26], the adhesion molecule of dendritic cells (DC-SIGN) [27], TIM/TAM family [28], and glycosaminoglycans (GAGs) [29], are involved in the attachment and receptor interaction of flaviviruses in mammalian cells. Prohibitin has been identified as a receptor of DENV in *Ae. albopictus* C6/36 and *Ae. aegypti* CCL-125 cells [30]. Host factors that mediate viral replication and assembly, as well as those involved in host immunity, are crucial for flavivirus infection. For instance, the transmembrane protein 41B is recruited to flavivirus RNA replication complexes to facilitate viral replication and acts as a pan-flavivirus host factor [31]. The src family kinase c-Yes is a cellular protein involved in WNV assembly and egress in HEK 293 cells [32]. A member of the human immunophilin family, cyclophilin A, is essential for the replication of yellow fever virus in BHK-21 cells [33]. The restricted infection of ISFs in mammals could be attributed to mismatches with cell receptors or other factors essential for viral replication or assembly.

ISFs affect the replication or transmission of arboviruses in both positive and negative ways [34]. Pre-infection of *Ae. aegypti* with both Phasi Charoen-like virus and Humaita Tubiacanga virus enhances the ability of mosquitoes to transmit DENV and ZIKV by blocking the downregulation of mosquito histone protein H4 [13]. Pre-infection and co-infection with cell-fusing agent virus (CFAV) significantly enhance DENV2 replication in Aag2 cells by increasing the expression of ribonuclease kappa. However, pre-infection with CFAV negatively interferes with both ZIKV and DENV1 infection in *Ae. aegypti* [35]. Nhumirim virus restricts the replication of ZIKV and DENV2 in *Aedes* mosquitoes, but not the alphavirus chikungunya virus, indicating that the inhibitory effect may be specific to flaviviruses [36]. The transmission of WNV was significantly reduced in *Cx. quinquefasciatus* when co-inoculated with Nhumirim virus, although the viral titers in saliva were not affected [34]. Prior infection of AEFV is detrimental to the replication of ZIKV and DENV2 in *Ae. albopictus* through activation of the JAK-STAT, Toll, or RNAi pathways. AEFV infection alone fails to activate the Toll pathway, while co-infection with ZIKV results in upregulation of the Toll pathway. This discrepancy may be attributed to either a temporally delayed response of the Toll pathway (due to different time points for detection) or an interaction between AEFV and ZIKV that synergistically induces Toll pathway activation. Beyond immune responses, other factors such as the specific tissue regions of viral infection and protein interactions between viruses also influence the co-infection process. For example, pre-infection with Palm Creek virus reduces mosquito susceptibility to oral infection with WNV, potentially due to competition for cellular resources, as both viruses share colonization sites within intestinal epithelial cells [37]. An alternative explanation for the interaction between the two viruses could be resource competition within co-infected cells [38]. This is consistent with the superinfection exclusion hypothesis, which suggests that cells infected with one virus are often resistant to subsequent infection by similar viruses [39, 40]. Exploring the precise mechanisms by which AEFV regulates arbovirus infection is essential for developing novel strategies to prevent arbovirus transmission.

## Conclusions

We isolated an AEFV strain from wild mosquito *Ae. albopictus* populations of Hainan Island, China. This AEFV Hainan strain is capable of replicating in *Aedes* mosquitoes but not in *Culex* mosquitoes, with the bottleneck being cell entry. Comfortingly, the infection risk of the AEFV Hainan strain was quite low in four mammalian cell lines due to the failure of either cell entry or viral replication. Furthermore, AEFV-carrying inhibits the replication of pathogenic flaviviruses such as ZIKV and DENV2 in *Ae. albopictus* by activating insect immunity. Our work indicates that the Hainan strain of AEFV poses little risk to human health and highlights its potential as an immune inducer to sabotage *Aedes* mosquito ability for viral transmission.

## Supplementary Information

Additional file 1: Table S1. Primers used in this study.

## Data Availability

The virus genome has been deposited in the National Center for Biotechnology Information with the accession number PQ223729.
