# Supplementary material for: Risk assessment of an Aedes flavivirus and its effect on pathogenic flavivirus replication in mosquitoes
Source: Parasit Vectors. 2025 Mar 5;18:88. doi: 10.1186/s13071-025-06711-4 (PMC11881423; doi:10.1186/s13071-025-06711-4)
Supplement: Supplementary file 1 — Additional file 1: Table S1. Primers used in this study. [file 13071_2025_6711_MOESM1_ESM.pdf]

Supplementary

Table S1 Primers were used in this study.

Primer name	Primer sequence (5' to 3')
Primers for PCR	
AEFV5'RACE-R	CAATGGCGGTACCATCTTCCGTTTCCA
AEFV3'RACE-F	TTGCATAGTCCTATTGCTACGGA
AEFV-1F	CCAACAAAAGAAGCGACTCCATCAT
AEFV-1R	AACCCCACCACCTCTCCATTTACAA
AEFV-2F	TCTCACGTCTGCCTCTTTGTTTGT
AEFV-2R	GACTTTTAGTGTTCTCGTCCGCTC
AEFV-3F	AGTAAATGAGACAGGTAAAGGTGA
AEFV-3R	CTACAGTAGATCCGTAGCAATAGG
Primers for qPCR	
AEFVNS5qPCR-F	GGTTTTGGAATCTCGTTGGAC
AEFVNS5qPCR-R	ACGCTTGGCTTCTTTTCTTTT
Ae.albo-actinqPCR-F	CGACCGTATGCAGAAGGAAA
Ae.albo-actinqPCR-R	TGGAAGGTGGATAGCGAGG
Ae.aegy-actinqPCR-F	CGTTCGTGACATCAAGGAAA
Ae.aegy-actinqPCR-R	GAACGATGGCTGGAAGAGAG
Cxq-actinqPCR-F	CGGGTATTGTGCTGGACTC
Cxq-actinqPCR-R	GCGACGTAGCACAGCTTCT
BHK21-actinqPCR-F	CAGGGTGTGATGGTGGGTATGG
BHK21-actinqPCR-R	AGTTGGTGACAATGCCGTGTTC
Vero-actinqPCR-F	GCGGGAAATCGTGCGTGAC
Vero-actinqPCR-R	ATGCCCAGGAAGGAAGGTTG
293T-actinqPCR-F	AAAACCTAACTTGCGCAGAAAACA
293T-actinqPCR-R	GGGCACGAAGGCTCATCATT
HeLa-actinqPCR-F	CTGGAACGGTGAAGGTGACA
HeLa-actinqPCR-R	AAGGGACTTCCTGTAACAACGCA
Zika-E-qPCR-F	GGGGAAACGGTTGTGGACTT
Zika-E-qPCR-R	CTGGGAGCCATGCACTGATA
DENV2-E-qPCR-F	CAGGCTATGGCACTGTCACGAT
DENV2-E-qPCR-R	CCATTTGCAGCAACACCATCTC
PIASqPCR-F	ACACTTGTGCCGAGCAATACC
PIASqPCR-R	AAACCGCAACTGAACCTGAATG
CactusqPCR-F	CGTGAGGCTTGCCAAACGAAC
CactusqPCR-R	CCTTCTGAAACGGGAGGGTGC
Dicer2qPCR-F	GAGCCGCAATCTAACCTACGA
Dicer2qPCR-R	CGTGCAAACAACGACCTGAA
Probes for FISH	
AEFVCPprobe-F	GGAACTGACGGAAATGGGAAA
AEFVCPprobe-R	TAATACGACTCACTATAGGGCAACGGAGACAACGAGAAGGC

F, forward primers; R, reverse primers.
